# Ectopic Overexpression of Porcine Myh1 Increased in Slow Muscle Fibers and Enhanced Endurance Exercise in Transgenic Mice

**DOI:** 10.3390/ijms19102959

**Published:** 2018-09-28

**Authors:** Jin Seop Ahn, Dong-Hwan Kim, Hee-Bok Park, Sang-Hyun Han, Seongsoo Hwang, In-Cheol Cho, Jeong-Woong Lee

**Affiliations:** 1 Biotherapeutics Translational Research Center, Korea Research Institute of Bioscience and Biotechnology, Daejeon 34141, Korea; ajsws@naver.com (J.S.A.); dhkim00@kribb.re.kr (D.-H.K.); 2 Department of Functional Genomics, University of Science and Technology, Daejeon 34113, Korea; 3 Subtropical Livestock Research Institute, National Institute of Animal Science, Jeju 63242, Korea; heebokp@korea.kr; 4 Educational Science Research Institute, Jeju National University, Jeju 63243, Korea; hansh04@naver.com; 5 Animal Biotechnology Division, National Institute of Animal Science, Wanju 55365, Korea; hwangss@korea.kr; 6 Animal Genetics and Bioinformatics Division, National Institute of Animal Science, Wanju 55365, Korea

**Keywords:** Myh1, quadriceps, slow muscle, transgenic mice

## Abstract

Myosin heavy chain (MyHC) isoforms consist of Myh7, Myh2, Myh1, and Myh4, which are expressed in skeletal muscle tissues during postnatal development. These genes influence the contraction–relaxation activity in skeletal muscles and are involved in determining muscle composition such as the proportion of fast-to-slow and/or slow-to-fast fiber types. Among them, Myh1 is associated with skeletal muscle contraction and is involved in both slow-to-fast and fast-to-slow transition. However, the muscle transition mechanism is not well understood. For this study, we first produced porcine *Myh1* transgenic (TG) mice to study whether the ectopic expressed porcine *Myh1* gene had any effects on muscle composition, especially on slow-type muscle components. Our results showed that the factors associated with slow muscles, such as Myh7, Myoglobin, Troponin (slow-type units), and cytochrome C, were highly expressed in the quadriceps muscles of *Myh1* transgenic mice. Furthermore, the ectopic porcine MYH1 protein was located only in the slow-type muscle fibers of the quadriceps muscles in *Myh1* transgenic mice. In physical endurance tests, *Myh1* transgenic mice ran longer and further on a treadmill than wild-type (WT) mice. These data fully supported our hypothesis that Myh1 is associated with slow muscle composition, with overexpression of *Myh1* in muscle tissues possibly being a new key in modulating muscle fiber types. Our study provides a better understanding of muscle composition metabolism, physical mobility, and genetic factors in muscle fatigue.

## 1. Introduction

Skeletal muscle is composed of slow (Type I) and fast (Type II) fibers, which convert chemical energy into energy for contraction and metabolism [1,2,3]. Slow-type fibers activate mitochondrial biogenesis and mainly use oxidative metabolism for energy products, which provide stable and continuous activity with fatigue resistance by providing a supply of adenosine tripohosphate (ATP). On the other hand, fast-type fibers show different characteristics, including lower mitochondrial content and oxidative metabolism, dependency on glycolytic metabolism, and ability to produce rapid activity with higher fatigue rates [4,5]. 

Adult skeletal muscles have four myosin heavy chain (MyHC) isoforms, which are namely Myh7 (Type I), Myh2 (Type IIA), Myh1 (Type IIX), and Myh4 (Type IIB). These show distinct expression patterns in troponin, myosin light chain, and sarcoplasmic calcium dependent ATPase [6,7,8]. Additionally, the MyHC isoforms are co-expressed in hybrid fibers, which are classified as Type I/IIA (includes Type IC or Type CI), Type IIA/IIX (includes Type IIAX or Type IIXA), and Type IIX/IIB (includes Type IIXB or Type IIBX) [9]. A recent study showed that the transcriptional coactivators, which are namely PGC-1α and PGC-1β, interact directly with the muscle fiber transition [10,11]. PGC-1β is a potential mediator in highly oxidative slow fibers containing Type IIX [10]. Additionally, different expression patterns in MyHC isoforms, which appear in fast-to-slow or slow-to-fast fiber transition, are caused by neuromuscular activity, hormones, and mechanical loading and unloading [12]. Furthermore, another specific fiber isoform consists of three types of troponin subunits, which are namely tropomyosin, myosin light chain, and various calcium regulatory proteins [13]. 

Myh1 contributes to the fast fiber twitch by glycolytic metabolism and acts as an intermediator between Myh2 and Myh4. Furthermore, it has been reported that Myh1 is often associated with an oxidative mechanism that is similar to that of type I and IIA [14] and plays a role in the transformation of a muscle type from type IIB to IIA [15]. In particular, pig Myh1 is one of the candidate genes that determines meat quality traits [16], which is associated with muscle compositions [17]. However, the correlation between Myh1 and muscle composition is still unclear. 

To understand more specific roles and mechanisms of Myh1, we produced ectopic overexpressed porcine *Myh1* transgenic (TG) mice in order to first identify and characterize the roles of Myh1 in determining muscle composition. Our study showed that pig Myh1 is associated with the activation of the slow muscle components by increasing the expression level of slow muscular sarcomere-associated genes, which increases the overall physical endurance capacity. 

## 2. Results

### 2.1. Identification of Porcine Myh1 Gene

**Figure 1:**
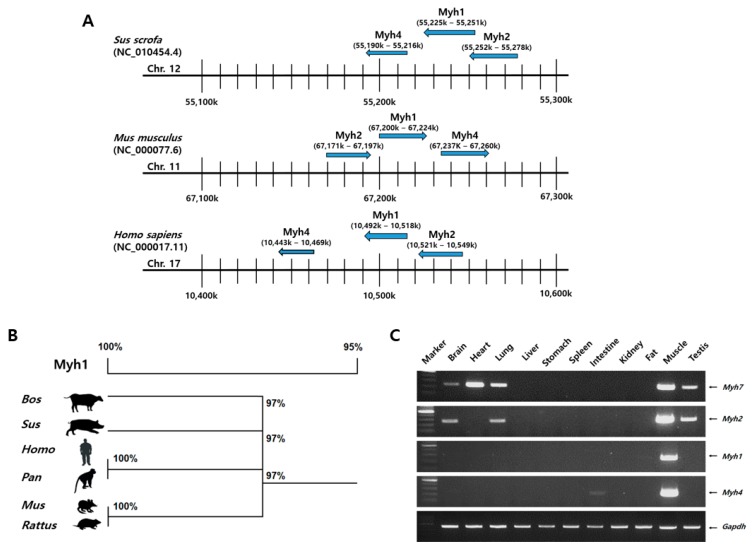
Identification of porcine *Myh1* gene. Multiple protein sequence alignment of *Myh1* gene in other species. (**A**) Myosin heavy chain gene clusters with species-specific loci. According to the NCBI annotations, Myh family genes that are associated with meat quality traits were located closely: *Sus scrofa* 11.1, *Homo sapiens* GRCh38.p12, and *Mus musculus* GRCm38.p6. (**B**) Homology and phylogenetic tree of *Myh1* gene. The number represents the open reading frame (ORF) homology percentage and difference between the ORF sequence in vertebrate species through an alignment tree using the NCBI database and the DNAMAN program. Bos: *Bos taurus* (NM 174117.1); Sus: *Sus scrofa* (NM 001104951.1); Homo: *Homo sapiens* (NM 005963.3); Pan: *Pan troglodytes* (XM 003953128.2); Mus: *Mus musculus* (NM 030679.1); Rattus: *Rattus norvegicus* (NM 001135158.1). (**C**) Comparisons of expression patterns of MyHC isoforms in pig tissue. RT-PCR was performed to identify the expression patterns in diverse pig tissues. Porcine *Gapdh* was used as an internal control.

To identify MyHC gene clusters which are associated with meat quality traits, we compared the species-specific loci on a chromosome using NCBI annotations: *Sus scrofa* 11.1, *Homo sapiens* GRCh38.p12, and *Mus musculus* GRCm38.p6 (Figure 1A). MyHC isoforms were located very closely on the *Sus scrofa* chromosome 12, *Mus musculus* chromosome 11, and *Homo sapiens* chromosome 17. We also identified the amino acid sequence of the porcine *Myh1* gene, before the interspecies homology analysis was conducted using the DNAMAN 8 program (Figure 1B). Identified data showed that the porcine *Myh1* gene had 97% homology with other species. We verified gene expression patterns of the MyHC family in various porcine tissues using reverse transcription polymerase chain reaction (RT-PCR), and the data showed that *Myh7* and *Myh2* are expressed in the brain, lung, muscle, and testis. Furthermore, *Myh7* was highly expressed in the heart, while *Myh*4 was highly expressed in the muscle but had lower levels of expression in the intestine. However, *Myh1* showed a specific expression pattern in the muscle tissue (Figure 1C). 

### 2.2. Establishment of Ectopic Overexpression of Porcine Myh1 in Mice 

**Figure 2:**
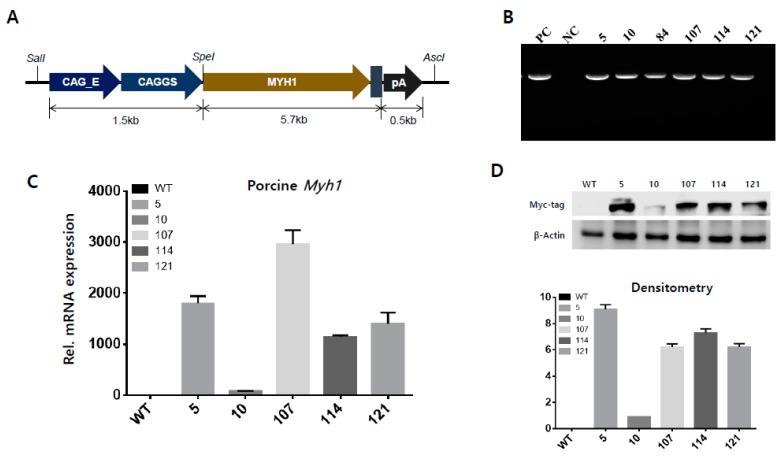
Development of porcine *Myh1* gene transgenic mice and confirmation of expression patterns. (**A**) Construction of pCAGGS-Myh1-Myc vector. The *Myh1* gene was inserted behind the CAG promoter and we subsequently followed Myc to use this for protein detection and poly A. The constricted vector was prepared for use by microinjection into mouse zygotes. (**B**) Conformation of transgene by genotyping. Tail-tips were cut from litters and genomic DNA was extracted. PC: positive control; NC: negative control; number: litter number. (**C**) Relative expression levels of porcine *Myh1* gene by qPCR. Each group was normalized by porcine GAPDH primer sets. (**D**) Analysis of western blot. A Myc-tag was used to detect protein expression and the relative protein level was quantified using Image J software. PC: positive control; NC: negative control; WT: wild-type mice; numbers such as 5, 10, 107, 114 and 121: line number of TG.

To verify the effects of porcine *Myh1* overexpression in skeletal muscle, we constructed a pCAGGS-MYH1-Myc tagging vector to produce TG mice. Briefly, Myc tailed-porcine *Myh1* was inserted between the enhancer/chicken β-actin (CAG) promoter and poly A (Figure 2A), before each of the sites that were inserted in the backbone vector were confirmed by sequencing analysis. To produce *Myh1* over-expressed mice, the constructed vector was microinjected into mouse zygotes and then, they were transferred into post-mothers. Transgenic mice were identified by genotyping (Figure 2B). To analyze the quantification of the transgene from the founder (F0) mice, the expression levels of transgene were analyzed by real-time PCR (qPCR) (Figure 2C) and a Myc-tag antibody was used for western blot analysis (Figure 2D). Although #107 showed the highest mRNA expression levels, protein levels were much higher in #5 than #107. Therefore, we selected #5 mouse as a breeder for the following studies. 

### 2.3. Effects on Slow Fiber Muscle in Myh1 TG Mice

**Figure 3:**
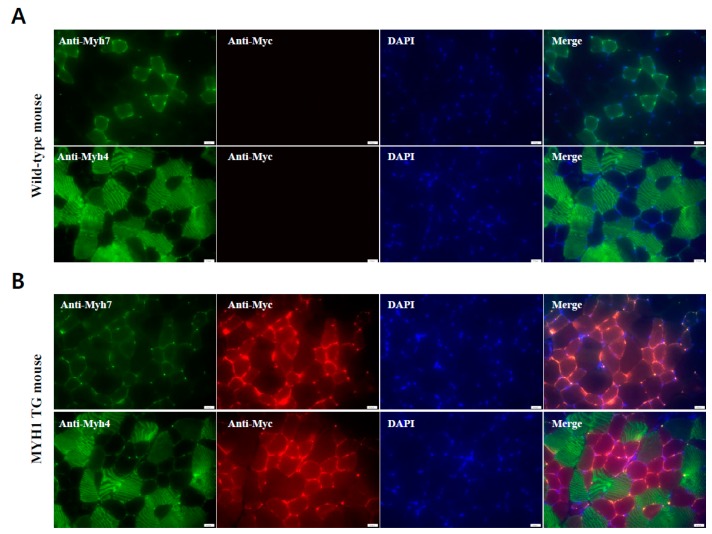
**Localization of ectopic overexpressed *Myh1* gene between slow and fast muscle fibers.** Analysis of Immunohistochemistry in quadriceps muscles of (**A**) WT and (**B**) Myh1 TG mice. Anti-MYH7 and anti-MYH4 were detected by green fluorescence, whereas anti-Myc was detected by red fluorescence. 4’,6-diamidino-2-phenylindole (DAPI)-stained nuclei. The scale bar represents 20 µm.

We investigated the expression of ectopic porcine MYH1 protein using immunohistochemistry (IHC) (Figure 3A,B). Surprisingly, Myc-tailed porcine MYH1 protein is localized with Myh7, which is a specific type of slow-twitch muscle.

**Figure 4:**
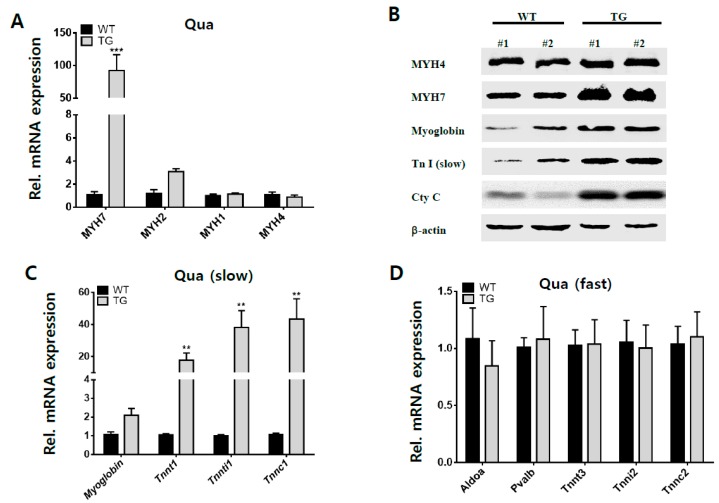
Expression of slow and fast muscle-associated genes in quadriceps muscles. (**A**) Myosin heavy chain gene expressions by qRT-PCR. *Myh7* is represented as a slow muscle type, whereas *Myh2*, *Myh1*, and *Myh4* are fast muscle types. (**B**) Comparisons of protein levels associated with slow muscle types between WT and TG in quadriceps muscle by western blotting. Overall, slow muscle type associated proteins were up-regulated in quadriceps muscles of TG mice. (**C**) Analysis of slow muscle-associated genes expression by qRT-PCR. (**D**) Analysis of fast muscle-associated genes expression by qRT-PCR. These data were from four-month-old wild-type (*n* = 4) and transgenic mice (*n* = 5), which were normalized according to the amount of mouse *Gapdh* mRNA and expressed relative to the corresponding value of the wild-type mice. Data are expressed as the means ± SEM for three independent replicates and multiple *t*-tests were used for the statistical analysis. * = *p* < 0.05; ** = *p* < 0.01; *** = *p* < 0.01.

To verify the effect of overexpressed *Myh1* in skeletal muscle, we analyzed the expression patterns of mouse myosin heavy chain genes (*Myh7*, *Myh2*, *Myh1*, and *Myh4*) in quadriceps muscles (Figure 4A). Skeletal muscles are composed of heterogeneous fibers. Myosin heavy chain isoforms are known as muscle fiber specific molecular markers [2,18]. The slow oxidative marker, *Myh7*, was significantly increased by approximately 100 times in terms of mRNA expression, whereas the mRNA of the fast oxidative marker, *Myh2*, was increased by approximately three times compared to wild-type mice. However, other fast glycolytic markers, *Myh1* and *Myh4*, showed no differences in mRNA expression in both TG and wild-type (WT) mice. To obtain further detail, we analyzed the oxidative muscle types, *Myh1*, *Myh7*, and *Myh4* by western blot. MYH7 protein was increased in TG group as shown in Figure 4A, but the MYH4 protein level was the same in WT and TG mice (Figure 4B). In addition, TG mice showed apparently enhanced expression levels of myoglobin, troponin I (slow) proteins, and mitochondrial oxidative cytochrome c enzyme, which have important functions of muscle oxidation as well as essential features of being a type I muscle composed of slow fibers (Figure 4B). These results clearly demonstrate that there is a greater proportion of slow myofibers in the skeletal muscle of the TG mice, which was driven by the overexpression of *Myh1*. Moreover, qPCR analysis revealed an increased expression level of slow-type genes, such as *Myoglobin*, *Tnnt1*, *Tnni1* and *Tnnc2* (Figure 4C). On the other hand, there was no significant difference in fast-type genes, such as *Aldoa*, *Pvalb*, *Tnnt3*, *Tinni2* and *Tnnc2*, between TG and WT mice (Figure 4D). 

### 2.4. Effect of Ectopic Expressed Porcine Myh1 to Muscle Type Formation

**Figure 5:**
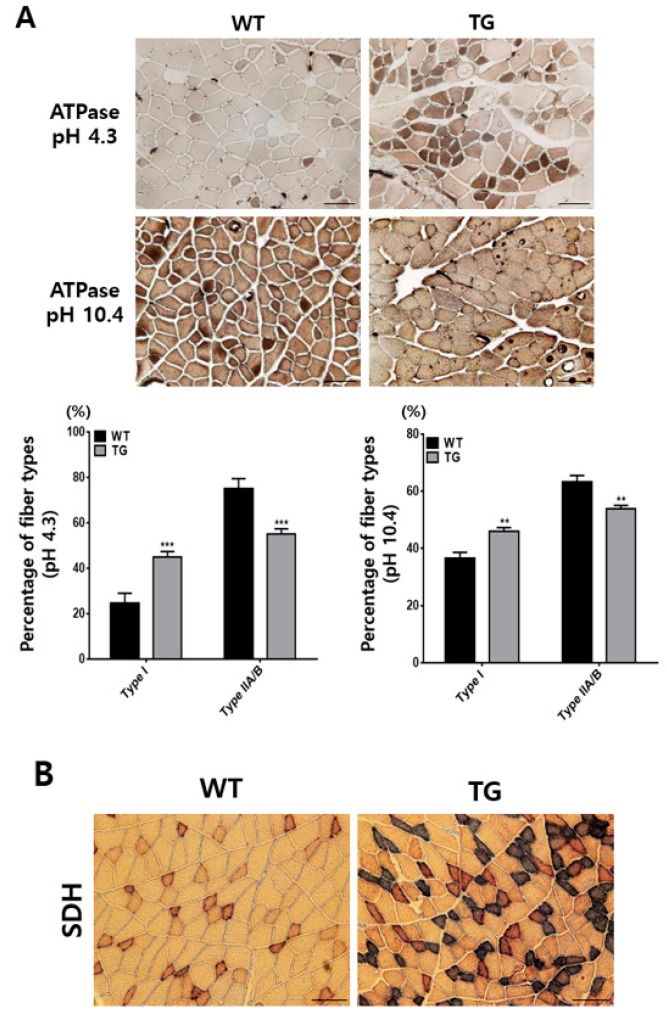
Comparisons of quadriceps muscle type in mice. (**A**) ATPase staining and morphometric analysis of quadriceps in WT and TG mice. When pH is 4.3, type 1 fibers show brown and type 2 fibers show a beige/white color: type I fibers, slow muscle type; WT vs. TG, 24.8% vs. 45%, whereas type II fibers, fast muscle type; WT vs. TG, 75.2% vs. 55%. When pH is 10.4 data, type 1 fibers show beige/white and type 2 fibers show a brown/black color: type I fibers, slow muscle type; WT vs. TG, 36.6% vs. 46.1%, whereas type II fibers, fast muscle type; WT vs. TG, 63.4% vs. 58.9%. (**B**) Mitochondria oxidation analysis by succinate dehydrogenase (SDH) staining. More mitochondrial oxidation (slow-type muscle fibers) is shown by a dark color. Data are expressed as the means ± SEM for three independent replicates and multiple *t*-tests were used for the statistical analysis. ** = *p* < 0.01 and *** = *p* < 0.01. The scale bar represents 100 µm.

To verify the effect of *Myh1* on muscle types in TG mice, the ATPase stain was used (Figure 5A). With a pH of 4.3, the brown color sections (type I fibers, slow muscle type; WT vs. TG, 24.8% vs. 45%) were shown more compared to the white or beige color sections (type II fibers, fast muscle type; WT vs. TG, 75.2% vs. 55%), which were seen less in TG mice compared to the WT. Furthermore, when the pH was 10.4, Type I fibers (beige or white color section; WT vs. TG, 36.6% vs. 46.1%) were more clearly shown, whereas type II fibers (brown color section; WT vs. TG, 63.4% vs. 58.9%) were not seen in the TG. In the same manner, succinate dehydrogenase (SDH) staining also showed that the muscle cells in the TG showed more mitochondria oxidation, with an increased proportion of slow muscle fibers (dark color section) (Figure 5B).

### 2.5. Enhanced Physical Performance in Myh1 TG Mice

It is known that type I muscle highly expresses *Myh7* and is a crucial muscle type in determining muscle fatigue resistance, because it contributes to muscle oxidation [19]. Additionally, physical performance, such as running, can be increased by slow-type muscles [19,20]. To prove that *Myh1* can affect physical performance, we compared the exercise performance of WT and TG mice on a treadmill until exhaustion. The running time and distance were significantly increased in the TG group: WT vs. TG, 865.7 ± 57.5 m vs. 1864 ± 448.6 m in running distance and 88.3 ± 4.7 min vs. 155 ± 26.1 min in running time (Figure 5A; see also Videos S1–S3). TG mice were able to run for an hour longer, which translated to a greater distance of 1000 m compared to WT mice (*n* = 3). Thus, physical endurance is one of the physiological conditions of muscle transition, and based on this treadmill data, *Myh1* could have direct effects on slow muscle fibers.

## 3. Discussion

In this study, we first demonstrated the physical and functional properties of the porcine *Myh1* gene. The overexpression of *Myh1* affected the up-regulation of factors that are associated with slow muscle fibers, and exercise endurance was increased in the ectopic overexpressed *Myh1* TG mouse, which represents muscle oxidative capacity. The characterization of ectopic overexpressed *Myh1* TG mice demonstrated that this overexpression resulted in an increase in the number of slow myofibers and mitochondrial biogenesis in quadriceps muscles. 

Skeletal muscles contain heterogeneous myofibers, which are slow- and fast-type fibers, in mammalian skeletal muscles [3,8]. In particular, slow-type fibers, which highly express *Myh7*, have high oxidative and abundant mitochondrial content, whereas fast-type fibers show powerful contraction properties and have smaller mitochondrial content than slow-type fibers. Furthermore, slow-twitch type I is an abundant isoform in the ventricles during the fetal life that expresses β-myosin heavy chain protein, which has a distinct rate of converting ATP for energy metabolism [21]. Our data showed that the overexpressed *Myh1* strongly increased *Myh7* mRNA and protein levels. Furthermore, the overexpressed *Myh1* induced other slow muscle-associated genes. To avoid any uncertainty over whether the effects resulted from the ectopic overexpressed porcine *Myh1* or the intrinsic mouse *Myh1*, porcine Myh1 was detected by Myc-tag on western blot analysis and IHC. In particular, the ectopic overexpressed porcine Myh1 was co-localized with Myh7, which is expressed in slow-type muscle fibers. These effects appear to be immediate consequences of *Myh1* activation, which is caused by transcriptional coactivators that are involved in slow muscle fiber functions and mitochondrial biogenesis in mice [19,22].

**Figure 6:**
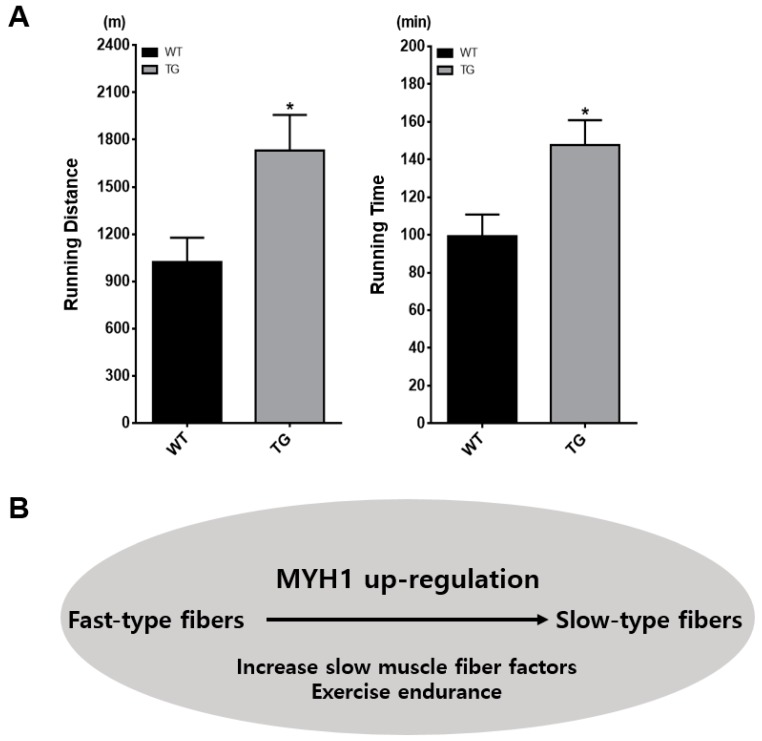
Enhanced physical exercise with Myh1. (**A**) Exercise endurance test in WT and TG mice on a treadmill. Sixteen-week-old male mice were subjected to a treadmill with foot-shock (*n* = 3 for each group). Running time and distance were checked until mice were exhausted. Data are expressed as the mean ± SEM for three mice in each group and multiple *t*-tests were used for the statistical analysis. * = *p* < 0.05. (**B**) Diagram of Myh1 function in skeletal muscle.

Although several genes were studied for muscle development, the role of genetic regulation in the development of specific fiber types is still unclear. Our data showed that slow muscles that are associated with *Myoglobin* and slow sarcomeric genes, *Tnnt1*, *Tnnti1* and *Tnnc1*, were highly expressed in TG mice, but that there was no significantly difference in the fast sarcomeric genes, *Aldoa, Pvalb, Tnnt3, Tnni2* and *Tnnc2*. ATPase and SDH staining data showed that there was a greater number of slow muscle fibers in TG mice. Therefore, the results showed that *Myh1* might cause an increase in the number of slow fibers through genetic factors. In the same manner, the physical performance data also proved that increased *Myh1* had an effect on muscle oxidative endurance, which is associated with slow muscle type fibers. This supports our model (Figure 6B), which hypothesized that *Myh1* can directly increase the number of slow muscle fibers.

Various signaling pathways, such as myocyte enhancer factor-2 (MEF2), histone deacetylase, calcineurin (CaN)/nuclear factor of activated t-cells (NFAT), calcium/calmodulin dependent protein kinase, Ras/mitogen-activated protein kinase and insulin-like growth factors, are involved in skeletal muscle remodeling, which are regulated by gene modulation and the selection of MyHC isoforms [8,23]. In a mammalian model, it was reported that the *Myh1* gene is related to muscle development and is significantly associated with meat quality, such as meat color, intramuscular fat (IMF), marbling, and moistures according to genome-wide association studies (GWAS) and quantitative trait loci (QTLs) [16,24]. A higher fast-type muscle ratio in the body is associated with poor meat quality and physical metabolism. However, the increase in slow-type muscle could reduce several problems, as mentioned above [25].

In conclusion, our study indicates that the *Myh1* gene could be a key modulator that regulates slow muscle fiber transition and physiological conditions, such as endurance and running capacity. This work has deepened the understanding of muscle transition metabolism and genetic basis in muscle fatigue.

## 4. Materials and Methods

### 4.1. Use of Mice and IACUC Commitment

All mice (C57BL/6) used in this study were kept at the Korea Research Institute of Bioscience and Biology (KRIBB) animal facility under pathogen-free conditions in a temperature-controlled climate at 22 ± 2 °C under a 12 h light/dark cycle. All animal experiments were approved by the Institutional Animal Care and Use Committee of the KRIBB (approval number and date: KRIBB-AEC-16077, 01 April 2016) and were performed in accordance with the Guide for the Care and Use of Laboratory Animals published by the U.S. National Institutes of Health (8th edition, 2011). All animals had free access to food and water during the experiments.

### 4.2. Construct of Myh1 Overexpression Vector and TG Mice Production

For the convenience of cloning, the full coding sequences (CDS) of the porcine *MYH1* gene was divided into four parts and synthesized by Bioneer Co. (Daejeon, Korea), before the full-length *Myh1* CDS was inserted into the pCAGGS-EGFP-Puro vector, which was used for TG mice production (Figure 2A). Furthermore, in order to avoid any interactions between the intrinsic mouse *Myh1* and transient porcine *Myh1* in TG mice, a Myc-tagging sequence was appended to the 3’ end of the open reading frame (ORF) to detect the expression of recombinant protein directly through western blotting. TG mice were produced by microinjection. A total of 126 litters were genotyped and among them, six transgenic mice were confirmed by genotyping (Figure 2B). PCR analysis was used with the following primer sets to confirm the TG mice: for genomic DNA PCR, forward 5’-GAA GCA GAT CCA GAA GCT GGA-3’ and reverse 5’-CTC CCA TAT GTC CTT CCG AGT-3’. The PCR was run under the following conditions: 35 cycles of 95 °C for 30 s, 57 °C for 30 s and 72 °C for 30 s. PCR products were electrophoresed on 1% agarose gel and stained with an ethidium bromide replacement.

### 4.3. Analysis of Gene Expression

Total RNA was isolated from muscle tissues using TRIZOL reagent (Ambion Inc., Austin, TX, USA). According to the manufacturer’s protocol, RNA was treated with DNase I and reverse transcribed into cDNA using the TOPscript cDNA synthesis kit (Enzynomics, Daejeon, Korea). For cDNA synthesis, 5 µg of each RNA sample was incubated at 55 °C for 60 min and 95 °C for 5 min. Each cDNA was used as a template for PCR amplification in combination with specific primers (Table 1). Quantitative RT-PCR (qRT-PCR) was performed using the TOP-real qPCR kit (Enzynomics, Daejeon, Korea) and a Rotor-Gene Q thermal cycler (Qiagen, Valencia, CA, USA). PCR was performed for 40 cycles of 95 °C for 20 s, 60 °C for 20 s, and 72 °C for 20 s. The expression levels were normalized to those of endogenous GAPDH and the data were analyzed using the ΔΔ*C*_t_ method [26].

### 4.4. Western Blotting

Tissue protein lysates were prepared in RIPA buffer with a protease inhibitor cocktail (Roche Diagnostics, Basel, Switzerland). After this, extracted proteins were quantified using BSA protein assay reagent (Bio-Rad, Hercules, CA, USA). Then, proteins (30 µg) were processed on precast 12% SDS-PAGE and transferred when semi-dry onto a PVDF membrane at 200 mA for 2 h. After blocking with 5% skim milk in a tris-buffered saline with 0.1% tween 20 (TBST) solution, membranes were incubated overnight at 4 °C with the indicated specific primary antibodies (Table 2). Secondary antibodies were diluted 1:10,000 in the blocking solution and incubated for 1 h at room temperature. After this, a signal was developed using an enhanced chemiluminescence (ECL) reagent (GE healthcare, Buckinghamshire, UK) and detected by an LAS-3000 luminescent image analyzer system (Fujifilm, Tokyo, Japan).

### 4.5. Immunohistochemistry

Quadriceps muscles were dissected and fixed in 4% formaldehyde overnight, before being embedded in paraffin. Serial sections of the embedded samples were de-paraffinized in xylene and re-hydrated in serial diluted alcohol. After this, the sections were permeabilized in 0.1% Triton X-100 and left for 1 h in the serum containing the blocking solution. MYH7 (Santa Cruz, Dallas, TX, USA) and MYH4 (Santa Cruz, Dallas, TX, USA) antibodies were diluted 1:100 in PBS and incubated overnight at 4 °C. After washing with PBS containing Tween 20, the sections were incubated for 1 h with the appropriate fluorescent-conjugated secondary antibodies and visualized under the fluorescence microscope. 4’,6-diamidino-2-phenylindole (DAPI) was used for the nuclei stain. 

### 4.6. ATPase and Succinate Dehydrogenase Staining

To identify which muscle types comprise the quadriceps in WT and TG mice, ATPase analysis was conducted using a ATPase Stain kit (#30-30125LY, Bio-Optica, Milano, Italy) following the manufacturer’s instructions. Briefly, cryostatic muscle sections (7 µm) of mice quadriceps were warmed up at room temperature for 10 min. The sections were incubated in each of the acetate buffers (pH of 4.3 and 10.4) for 10 min and Adenosine 5’-triphosphate solution for 30 min at 37 °C. After this, they were incubated in a cobalt chloride solution and an ammonium sulfide solution before being dehydrated and cleaned. Furthermore, in order to distinguish which muscles of WT or TG had oxidized to a greater degree, the quadriceps were stained by succinate dehydrogenase (SDH). Cryostatic muscle sections (7 µm) of mice quadriceps were warmed up and incubated in an SDH staining solution that consisted of 50 mM sodium succinate (#S2378, Sigma-Aldrich, St. Louis, MO, USA), 50 mM phosphate buffer (Welgene, Daegu, Korea) and 0.5 mg/mL nitroblue tetrazoliumand (#N5514, Sigma) for 40 min at 37 °C. All slides were visualized using an Olympus DP73 microscope (Olympus, Center Valley, PA, USA). 

### 4.7. Physiological Studies

To test exercise endurance, the treadmill was utilized according to the method described in a previous study [19]. Before the exercise performance test, the mice were trained to be familiar with the treadmill (Iwoo Scientific Corporation, Seoul, Korea) with a 10 min run at 7 m/min once a day for two days. The exercise test regimen was 7 m/min for the first 20 min, which subsequently was increased to 10 m/min for 40 min. After 60 min of running, the speed was increased by 1 m/min at 15-min intervals until the mice were exhausted, with a maximum speed of 18 m/min. Exhaustion was defined as when the mice were unable to avoid the repetitive electrical foot-shock.

### 4.8. Statistical Analysis

All experiments were repeated at least three times and statistical analyses were performed using multiple *t*-tests using the GraphPad Prism 6.02 software. The results with *p* < 0.05 were considered to be significant. The results are expressed as the mean ± SEM.

## Figures and Tables

**Table 1:** List of primer sequences.

Gene	Primer Sequence (5’-3’)		Size (bp)
	Forward	Reverse	
*Myh7* (mouse)	AGTCCCAGGTCAACAAGCTG	TTCCACCTAAAGGGCTGTTG	145
*Myh2* (mouse)	AGTCCCAGGTCAACAAGCTG	GCATGACCAAAGGTTTCACA	130
*Myh1* (mouse)	AGTCCCAGGTCAACAAGCTG	CACATTTTGCTCATCTCTTTG	113
*Myh1* (porcine)	CTTCACTGGCGCAGCAGGT	AGATGCGGATGCCCTCCA	256
*Myh4* (mouse)	AGTCCCAGGTCAACAAGCTG	TTTCTCCTGTCACCTCTCAACA	100
*Myoglobin* (mouse)	GCAAGGCCCTGGAGCTCTTC	GCTTGGTGGGCTGGACAGTG	100
*Tnnt1* (mouse)	CCCCCGAAGATTCCAGAAGG	TGCGGTCTTTTAGTGCAATGAG	154
*Tnni1* (mouse)	ATGCCGGAAGTTGAGAGGAAA	TCCGAGAGGTAACGCACCTT	140
*Tnnc1* (mouse)	GCGGTAGAACAGTTGACAGAG	CCAGCTCCTTGGTGCTGAT	103
*Aldoa* (mouse)	GGCAACACCCAGCAACAGAC	TCATCTGCAGCCAGGATGCC	158
*Pvalb* (mouse)	ATCAAGAAGGCGATAGGAGCC	GGCCAGAAGCGTCTTTGTT	252
*Tnnt3* (mouse)	GGAACGCCAGAACAGATTGG	TGGAGGACAGAGCCTTTTTCTT	104
*Tnni2* (mouse)	AGAGTGTGATGCTCCAGATAGC	AGCAACGTCGATCTTCGCA	170
*Tnnc2* (mouse)	ATGGCAGCGGTACTATCGACT	CCTTCGCATCCTCTTTCATCTG	72
*GAPDH* (mouse)	TGAAGGTCGGTGTGAACGG	CGTGAGTGGAGTCATACTGGAA	150
*GAPDH* (porcine)	CCTTCATTGACCTCAACTACAT	CCAAAGTTGTCATGGATGACC	400

**Table 2:** List of antibodies.

Antibody	Number	Source
Myc-Tag	#2276	Cell Signaling
MYH4	SC-168672	Santa Cruz Biotechnology
MYH7	SC-53089	Santa Cruz Biotechnology
Myoglobin	SC-8081	Santa Cruz Biotechnology
Troponin I-SS	SC-8119	Santa Cruz Biotechnology
Cytochrom C	SC-13156	Santa Cruz Biotechnology
β-actin	#4970	Cell Signaling

## Supplementary Materials

Supplementary materials can be found at http://www.mdpi.com/1422-0067/19/10/2959/s1. Video S1. Running performance 30 min later: This video shows the running exercise on a treadmill with foot-shock. The left lane represents TG mice and the right lane contains wild-type mice at five months old after 30 min of running. Video S2. Running performance 90 min later: This video shows the running exercise on a treadmill with foot-shock. The left lane represents TG mice and the right lane contains wild-type mice at five months old after 90 min of running. Video S3. Running performance 140 min later: This video shows the running exercise by a treadmill with foot-shock. The left lane represents TG mice at five months old after 140 min of running. Wild-type mice (right lane) were carried off the treadmill when they were exhausted.
